# Characteristics of functionalized nano-hydroxyapatite and internalization by human epithelial cell

**DOI:** 10.1186/1556-276X-6-600

**Published:** 2011-11-23

**Authors:** Zhao Yan-zhong, Huang Yan-yan, Zhu Jun, Zhu Shai-hong, Li Zhi-you, Zhou Ke-chao

**Affiliations:** 1 Medical Experiment Center in the Third Xiangya Hospital, Central South University, Changsha 410013, China; 2 State Key Laboratory of Powder Metallurgy, Central South University, Changsha 410083, China; 3 Research Center for Medical Material and Instruments, Central South University, Changsha 410013, China

**Keywords:** hydroxyapatite nanoparticles, arginine; europium, dope, cellular internalization

## Abstract

Hydroxyapatite is the main inorganic component of biological bone and tooth enamel, and synthetic hydroxyapatite has been widely used as biomaterials. In this study, a facile method has been developed for the fabrication of arginine-functionalized and europium-doped hydroxyapatite nanoparticles (Arg-Eu-HAP). The synthesized nanoparticles characterized by transmission electron microscopy, X-ray diffractometry, Fourier transform infrared, and Zeta potential analyzer. Its biological properties with DNA binding, cell toxicity, cell binding and intracellular distribution were tested by agarose gel electrophoresis assay, flow cytometry, and fluorescence microscope and laser scanning confocal microscope. The synthesized Arg-Eu-HAP could effectively bind DNA without any cytotoxicity and be internalized into the cytoplasm and perinuclear of human lung epithelial cells.

## Introduction

To date, one of the main barriers for gene therapy to achieve substantial breakthrough is probably due to the lack of high efficacy and safe gene delivery vector. The death of several clinical trials with viral-based gene delivery systems, especially the one using a retrovirus system, leads to more concerns for the future of gene therapy. The US Food and Drug Administration had suspended gene therapy trials [1,2]. In recent years, some nonviral-based gene delivery systems, such as functional cationic polymers [3-5] and nano-carriers [6-8], circumvent some of the problems occurring with viral vectors such as endogeneous virus recombination, oncogenic effects, and unexpected immune response, but their gene transfer efficiency is inferior to viral vectors. In addition, the cytotoxicity of cationic polymers is an essential problem in the polyplex-based gene transfer field. Therefore, to develop a novel gene delivery system with safe, non/low-toxic, non-immunogenicity, and easy-assemblage has recently received intensive attention.

Among nanoparticles with different materials composition, inorganic nanoparticles composed of calcium phosphate have numerous advantages including ease of synthesis, control of physicochemical properties, strong interactions with their payload, and biocompatibility. As the main inorganic component of biological bone and tooth enamel, hydroxyapatite shows excellent biocompatibility and bioactivity [9,10]. It has been widely used as an implant biomedical material in orthopedic and dental treatments [11,12]. Moreover, hydroxyapatite nanoparticles (HAP) are low crystalline with highly active surfaces and used as carrier in drug delivery systems as well as for protein separation as an absorptive material [13,14]. Interestingly, HAP can inhibit some cancer cells growth [15]. Our previous study reported [16] that HAP-incorporating pEGFP-N_1 _are able to deliver DNA into gastric cancer cells without any significant cytotoxicity, which transfer efficiency of is equal to 50% of liposome's under the equivalent conditions. Tan [17] discovered that after being modified by protamine, gene transfer efficiency of HAP can be enhanced more times. Sun [18] successfully used HAP to delivery NT-3 gene into the cochlear neurons of guinea pig both *in vitro *and *in vivo*. The demonstrating HAP may be a potential effective and safe material as a gene delivery agent. However, the low gene transfer efficiency limits their applications.

Nanoparticles with well-defined inner and outer surfaces that can be easily functionalized for biological application have attracted intensive attention recently in biotechnological studies [19,20]. To optimize the efficacy in gene delivery, the authors conjugated the hydrophilic arginine with a guanidyl group onto the surface of HAP in a previous study [21]. The result demonstrated that arginine-modified HAP had good biocompatibility and gene binding property. Meanwhile, some research revealed that arginine with guanidyl group can facilitate the cellular uptake of nanoparticles [22], but the mechanism of their uptake is disputed [23]. These physicochemical properties of HAP that provide for intracellular penetration of drug molecules have great importance for gene delivery.

In this article, the authors report a facile method for the fabrication of arginine-functionalized and europium-doped hydroxyapatite nanoparticles (Arg-Eu-HAP). Almost nontoxic and more stable inorganic europium is selected as fluorescent bioimaging probes [24-27]. Europium doping was performed to enable photoluminescence of HAP. The characterization of physicochemical and photoluminescence properties of Arg-Eu-HAP were examined. Preliminary studies on gene binding, cell toxicity, and cell uptaking *in vitro *were carried out. The results suggest that Arg-Eu-HAP with unique biological properties make them suitable for the next research as a gene delivery agent.

## Materials and methods

### Experiment materials

Calcium nitrate, ammonium phosphate, arginine (Sigma Corporation, St. Louis, MO, USA), pEGFP-N1 plasmid (Wuhan Genesil Biotechnology Co., Ltd., Wuhan, China) and other materials were used in this research. All reagents were of the highest analytical grade available. Cell culture media, fetal bovine serum, was obtained from American Type Culture Collection (Rockville, Maryland, USA). Ham's F-12 medium with L-glutamine was purchased from Fisher Scientific (Logan, UT, USA). Trypsin-EDTA (×1) and Hank's balanced salt solution were purchased from Invitrogen (Carlsbad, CA, USA). Phosphate buffer salt solution (PBS) and penicillin-streptomycin were obtained from Sigma-Aldrich (Logan, UT, USA). Ultrapure deionized water was prepared using a Milli-Q system (Millipore, Bedford, MA, USA).

### Synthesis of Arg-Eu-HAP

Arg-Eu-HAP were synthesized by hydrothermal method. Aqueous solution with calcium nitrate Ca(NO_3_)_2_·4H_2_O and europium nitrate Eu(NO_3_)_3 _was added dropwisely into ammonium dibasic phosphate (NH_4_)_2_HPO_4 _and arginine solution, and then were completely stirring and the mole ratio of Ca/P should be 1.67. The reaction temperature should be 60°C. During the reaction, the solution pH was maintained at 9.5 by using ammonia solution or urea. After calcium and phosphate solution was stirred evenly, the solution was transferred into an autoclave. Then the reaction was continued under the set solution temperature until completion. At the end of the experiment, the solids were collected by centrifugation (10,000 rpm/min) and filtration and then were washed thoroughly by using ethanol and deionized water. The product was dried overnight at the vacuum condition.

### Characterization of Arg-Eu-HAP

The nanoparticles samples were characterized by a transmission electron microscope (JEOL., Tokyo, Japan) to analyze the nanoparticle crystalline appearance and the particle size, X-ray diffractometry to have phase analysis on Arg-Eu-HAP (Rigaku D-Max/2550VB+, Tokyo, Japan, Cu Ka radiation, *λ *= 1.54178 Å, 40 Kv, 30 mA), where the scanning angle and speed should apply 25° to approximately 55°, 2.4°/min, or 5° to approximately 75°, 5°/min and the Fourier infrared spectrometer is Nicolet Nexus470, KBr flaking. The excitation and emission spectra of Arg-Eu-HAP were determined by a RF-5301pc spectrofluorometer (Shimadzu Corporation, Nakagyo-ku, Kyoto, Japan).

### Zeta potential measurement of Arg-Eu-HAP

Under the condition of neutral pH value (pH = 7.4), British Malvern Instrument Corporation's (Malvern, UK) Zetasizer 3000 HS nano size and potential analyzer was used to measure the electrophoretic mobility of Arg-Eu-HAP, thus obtain the Zeta potential. Eight samples were taken respectively, sample measurement was repeated three times, and their mean value was taken.

### DNA binding of Arg-Eu-HAP

Plasmid DNA (1 μg) was mixed with the solution of Arg-Eu-HAP suspension at various HAP/DNA mass ratios (0:1, 10:1, 30:1, 50:1, 70:1, and 90:1) and allowed to incubated at room temperature for 20 min before loading into the agarose gel. The solution was centrifuged at 12,000 rpm/min for 10 min and then its supernatant was taken to have electrophoresis on 0.7% (*w/v*) agarose gel (80 V) for 45 min and stained with ethidium bromide for 10 min. The staining results were investigated under UV transilluminator.

### Cell toxicity of Arg-Eu-HAP

The cytotoxicity of Arg-Eu-HAP was evaluated using flow cytometry in human lung epithelial (A549) cell line. In brief, cells were seeded in six-well tissue culture plates at a density of 1 × 10^5 ^cells per well. Three different concentrations of samples (20, 100, 200 μg/mL) were added to cell culture wells. After the cells were exposed to nanoparticles for 4, 8, 24, or 48 h, the experiments were terminated by flow cytometry (ChemoMetec, Allerød, Denmark) and the manufacturer's instructions were followed.

### Cell binding and cellular internalization of Arg-Eu-HAP

To track the internalization of Arg-Eu-HAP, A549 cells were seeded in 12-well plates at 1 × 10^5 ^cells per well and incubated. Subsequently, cells were rinsed twice with serum media (F-12K without FBS, pH 7.0) and replenished with 1 mL serum-free media containing Arg-Eu-HAP at a final concentration of 30 μg/mL. After incubation for 2 h at 37°C, test samples were aspirated. Cells were then washed twice with ice-cold phosphate-buffered saline (PBS) before they were fixed with fresh 4% paraformaldehyde for 3 min at room temperature. Finally, the fixed cells were counterstained to visualize nuclei by 4',6-diamidino-2-phenylindole (DAPI) (Sigma-Aldrich). The intracellular localization of nanoparticles was visualized under a laser scanning confocal microscope (Bio-Rad MRC 1024, Tokyo, Japan) equipped with Argon (488 nm) and HeNe (543 nm) lasers.

### Statistics

All experiments were repeated at least three times, and the values are expressed as means ± standard deviations. Statistical analysis was performed using student's *t *test, with the significant level with a *p *value of less than 0.05.

## Results and discussion

### Synthesis of Arg-Eu-HAP

**Figure 1:**
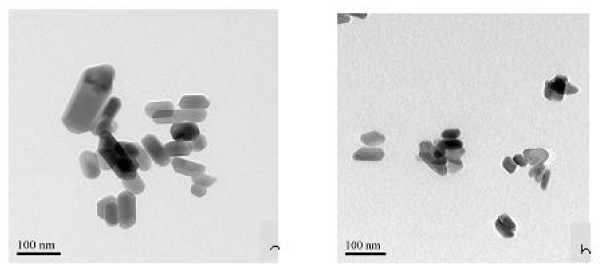
**TEM images of Arg-Eu-HAP crystal synthesized by hydrothermal method**. **(a) **Without amino acid; **(b) **with arginine

Figure 1 shows the TEM image of hydrothermal synthesized Arg-Eu-HAP, it can be perceived that unfunctionalized particles appear in short column shapes and the cross-sections of particles are even, approximately 50 to 100 nm. The lengthwise size of particles is in the size range of 50 to 200 nm (Figure 1a). After adding in arginine, the particles sizes reduce and turn to be grain shapes with the sizes of 50 to 80 nm (Figure 1b). During the process of synthesizing nanoparticles under the hydrothermal equilibrium conditions, the preferential growth direction of the HA crystal is [001]. Arginine's absorption of the seeded out HA crystal face selectively affects particles growth, the positive electron guanidyl group of arginine is able to have static effect with the negative electron hydroxyl exposed on the HA (001) face, resulting in intendancy of arginine to be absorbed on the (001) face of HA nanoparticles. Therefore, arginine's absorption hinders the solution-synthesized product to be separated out on the HA (001) face to the greater extent.

### Characterization of Arg-Eu-HAP

**Figure 2:**
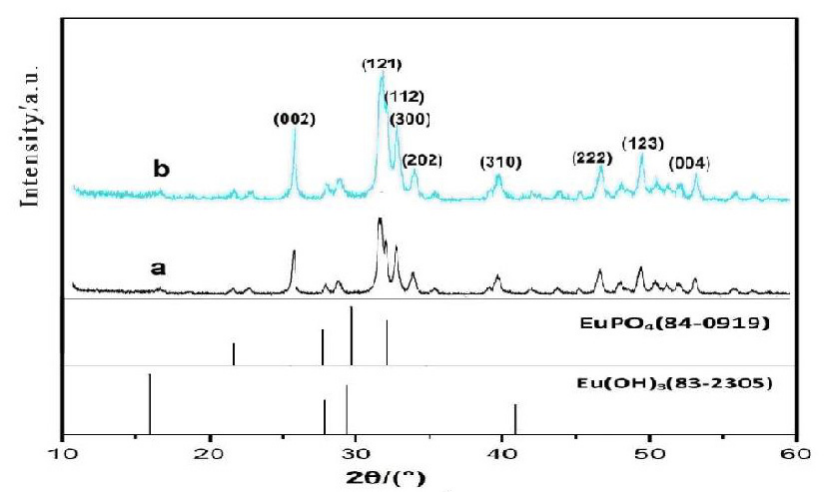
**XRD patterns of nanoparticles and Eu-doped nanoparticles**.

Figure 2 is the XRD graph of two groups of samples. It can be seen that all prepared nanoparticles' XRD graphs are similar. Their characteristic peaks are sharp and apparent, confirming that the resulting europium-doped HAP had the typical pattern of the pure HAP. All diffraction peaks could be assigned to the standard one (JCPDs 9-432). This demonstrates phenomenon as various direction sizes of the Arg-Eu-HAP samples shown in Figure 2 have concerted tendency and the solid particles' characteristics have strengthened.

**Figure 3:**
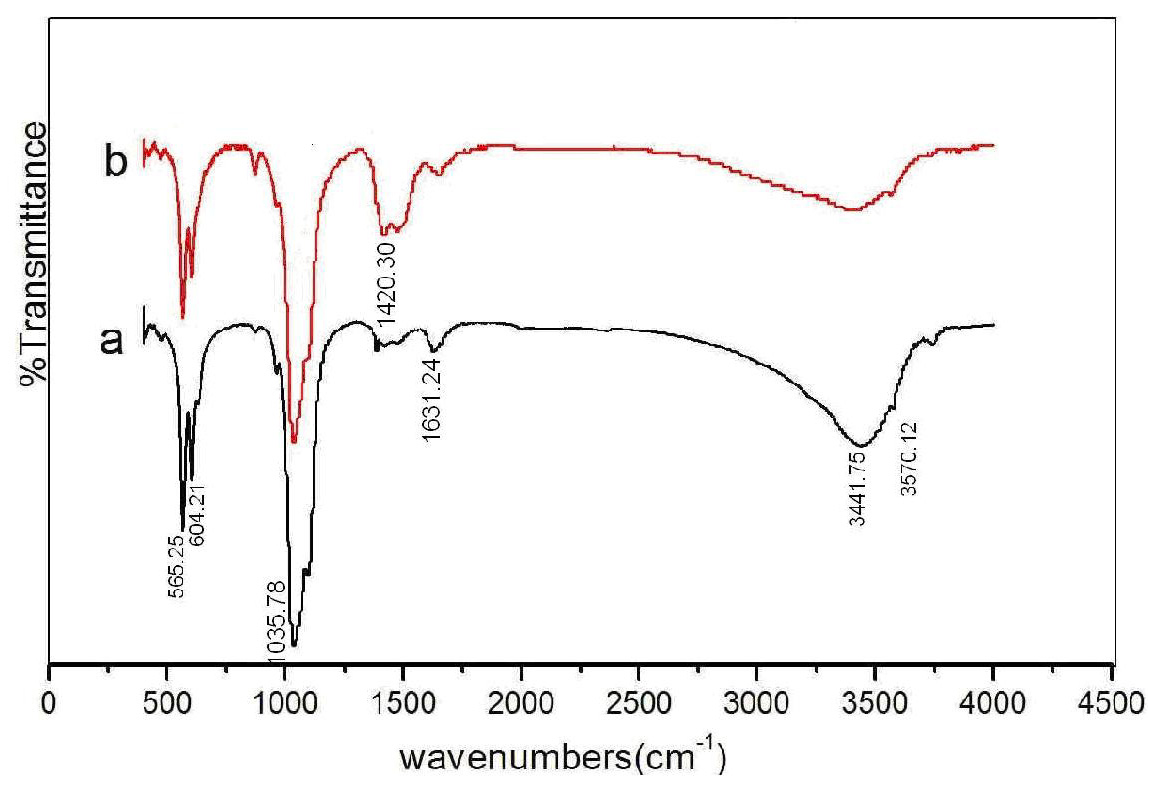
**FTIR spectra of arginine-functionalized nanoparticles: (a) without amino acid; (b) with arginine**.

The successful introduction of surface functionality was proved by Fourier transform infrared (FTIR; Figure 3), showed the infrared spectrometric waveforms of two sample groups are similar and the main peak positions of the graph are identical. The stronger peak lines occur at positions as 565.25, 604.21, 1,035.78, and 3,441.75 cm^-1^, and weaker or broader position peak lines occur at positions of 1,106.57, 1,420.30, 1,631.24, and 3,570.12 cm^-1^. The four vibration patterns corresponding peak positions of phosphate radicals in theory respectively are: ν_1 _peak at around 960 cm^-1^, ν_2 _peak at around 470 to 440 cm^-1 ^region, ν_3 _peak at 1,190 to 976 cm^-1 ^region, ν_4 _peak at 600 to 560 cm^-1 ^region. Therefore, the strong peaks at 565.25, 604.21, and 1035.78 cm^-1 ^and the weak peaks of 1,106.57 cm^-1 ^are generated by the phosphate radicals of HAP. The water molecule characteristic peaks in crystal lattice occur at the 3,550 to 3,200 cm^-1 ^region, thus the peaks of the 3,441.75 and 3,570.12 cm^-1 ^positions are the reflection of lattice water and hydroxy group (OH^-^). The characteristic peak at 1,631.24 cm^-1 ^is the vibration peak of H_2_O, indicating the surface of the solid samples absorbs a small amount of steam. The characteristic peak of amino group(-NH_2_) occurs in the 1,400 to 1,420 cm^-1 ^region and the 1,420.30 cm^-1 ^peak is perhaps the reflection of the absorption on HAP of the ammonium radical (NH_4_^+^) and amino acid residue derived from the raw material ammonium dibasic phosphate. For the added arginine sample, the intensity of this peak is somewhat strengthened, illustrating actual existence of amino acid residue.

**Figure 4:**
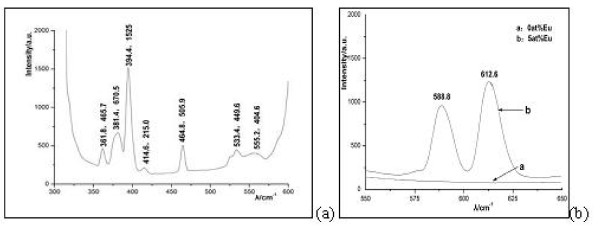
**Luminescence excitation (a) and emission (b) spectrum of europium-doped HAP**.

Europium ion (Eu^3+^) could be used as a luminescent probe in the bimolecular system. And Ca ions on the HAP surface could be replaced by the other metal cations with similar ionic radii, especially lanthanide ions. The formation of Eu-doped HAP could be confirmed by the luminescence study. The luminescence spectrum of Eu-doped HAP is shown in Figure 4. The emission spectrum with the excitation of 394.4 nm (Figure 4a) showed the luminescence at the wavelengths of 588.8 and 612.6 nm, which could be ascribed to ^5^D_0_-^7 ^F_1_, and ^5^D_0_-^7 ^F_2 _transitions of Eu, respectively. These emission effects could not be observed in the pure HA crystallites due to the absence of the featured Eu element. Thus, the presence of Eu in the HAP was confirmed. In addition, the more efficient emission with a maximum intensity at 612.6 nm is in the range of the emission filter chosen for the confocal microscopy. An excitation at 394.4 nm with the highest intensity is close to the visible range. However, another excitation peak was recorded at 464.8 nm, close to the available excitation wavelength in the confocal microscope. Observations on living cells are possible as this excitation wavelength is in the visible region.

### Zeta potential of Arg-Eu-HAP

**Figure 5:**
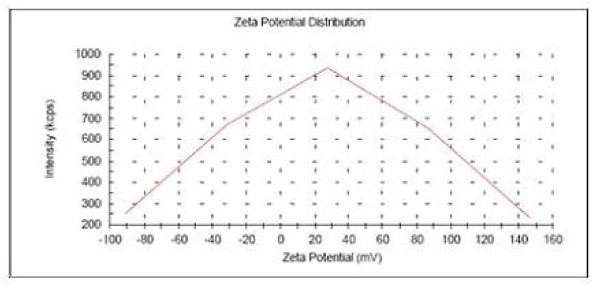
**Zeta potential curve of Arg-Eu-HAP at pH of 7.5**.

Figure 5 shows the Zeta potential of Arg-Eu-HA at the pH value of 7.5. Results suggested under the weak alkalescent condition (pH 7.5), the Zeta potential of Arg-Eu-HAP is (30.1 ± 6.3 mV) and unmodified HAP is (-10.6 ± 4.2 mV). This illustrates arginine surface functionalization of HA nanoparticles, cationic aminated functional groups increased its zeta potential value. This change comes from absorption of amino acids of amino acid residue on the Arg-Eu-HAP surface. In later researches, this substance is designed to be extracted from the aqueous solution medium synthesized from Arg-Eu-HAP and titrated to further discuss the hydrothermal crystalline behavior of HAP affected by arginine and the hidden mechanism of the surface electronic charge status.

### DNA binding of Arg-Eu-HAP

**Figure 6:**
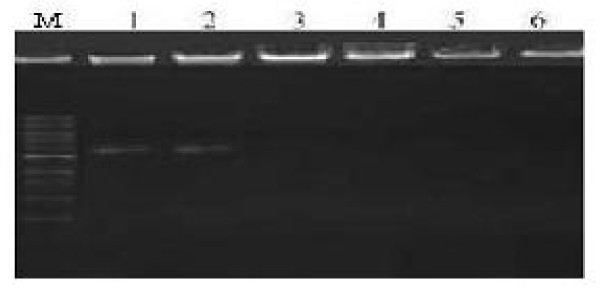
**Agarose gel electrophoresis of Arg-Eu-HAP/DNA complexes (*w*/*w *ratio)**. M, marker; L1, positive control; L2, 101:; L3, 301:; L4, 501:; L5, 701:; L6, negative control.

Due to arginine-functionalized on the HA nanoparticles, this can serve as the foundation for an effective enrichment of negatively charged DNA strands onto the positively charged nanoparticles surfaces. In this study, green fluorescence protein plasmid DNA was selected as a model DNA. Agarose gel electrophoresis demonstrated that Arg-Eu-HA could bind with DNA to form Arg-Eu-HA/DNA complexes. As shown in Figure 6, lane 1, naked plasmid DNA moved in the electric field, lanes 3 to 5, no uncomplexed pDNA was observed in the lane when mass ratios of Arg-Eu-HA to pEGFP-N1 plasmid are 30:1, 50:1, and 70:1, respectively, demonstrating DNA have fully bound with nanoparticles. The adsorption ratio is about 1 μg pEGFP-N1 pDNA per 30 μg HAP. The ultraviolet spectrometer 260-nm light absorption value measurement also proves the same result (data not shown).

### Cell toxicity of Arg-Eu-HAP

**Figure 7:**
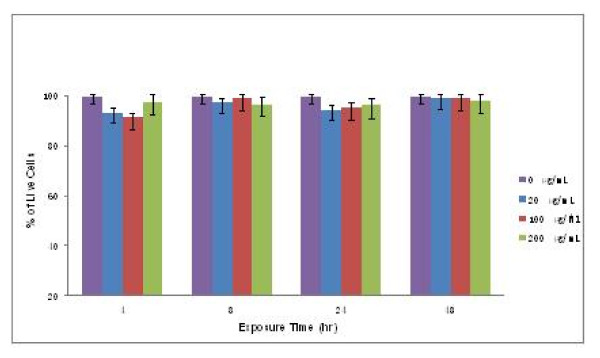
**Cell viability assay**. Cell viability assay showing the effect of varying concentrations of nanoparticles on growth inhibition of human lung epithelial (A549) cancer cells cultured *in vitro*. Results are reported as mean. There is no statistically significant difference between test groups and control groups (*p *< 0.05).

The effect of varying concentrations and exposure time of Arg-Eu-HAP on cell toxicity was evaluated using human epithelial lung cancer cell line (A549). The cell line was chosen as representative models of the various cellular environments that Arg-Eu-HAP are likely to interact with *in vivo*. Results showed that the studied Arg-Eu-HAP did not affect the cells survival in a concentration- and time-dependent manner. The cells exposed to nanoparticles survived well similar to those of the controls (Figure 7). Our data indicate that Arg-Eu-HAP is a potential gene carrier *in vitro*, and further preclinical and clinical development of this carrier for cancer gene therapy is warranted.

### Cellular uptake studies of Arg-Eu-HAP

Despite the unique advantages of HAP in biomedical applications, exploration of their interactions with biological systems remains at a very early stage. To effectively develop these systems for application, it is necessary to systematically delineate its functional properties about cellular uptake and interactions after arginine functionalized and europium doped. The majority of uptake studies *in vitro *have been performed in buffers devoid of protein. In physiological fluids, however, a protein corona could be formed on a particle surface and affect its interaction with cells [28,29]. We performed uptake studies in cell culture medium with free serum. Cellular uptake of Arg-Eu-HAP was investigated in A549 cell line.

**Figure 8:**
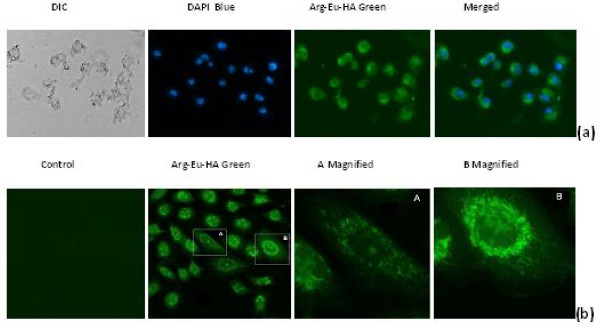
**Green emission and Laser scanning confocal microscope images**. **(a) **Green emission of the internalized Arg-Eu-HAP into the cells under fluorescence microscopy. Arg-Eu-HAP (green) were evident in the cytoplasm, nucleus were counterstained with DAPI dye (blue). Representative images of four different experiments are shown (magnification ×40). **(b) **Laser scanning confocal microscope images (magnification ×60, insert magnification ×252). No fluorescent light in the control cells can be detected.

In order to visualize the luminescence of the europium-doped nanoparticles and to demonstrate internalization in eucaryotic cells, several microscopic techniques were utilized. Figure 8a showed the fluorescence micrographs of DAPI-stained A549 cells after 2-h incubation with 30 μg/mL nanoparticles. It can be seen that most of the A549 cells incubated with Arg-Eu-HAP (green) were evident in the cytoplasm, nuclei were counterstained with DAPI dye (blue). These phenomena indicated a higher uptake of nanoparticles in A549 cells. The Laser scanning confocal microscope studies also verified the above results and showed that numerous luminescent nanoparticles were internalized within the A549 cells after 1 h and were observed in the cytoplasm of most cells (Figure 8b). Figure 8b (A magnified and B magnified) shows an accumulation of luminescent nanoparticles in the perinuclear areas of a cell on sections. No fluorescent light in the control cells can be detected (Figure 8b, control). Although the nanoparticles were detected throughout the endoplasm, no evidence of HAP entering the cell nucleus could be found from microscopy images in our study.

## Conclusions

In conclusion, nontoxic Arg-Eu-HAP have been prepared and characterized *in vitro *by various physicochemical means. As arginine surface functionalization changes HAP surface electron, its Zeta potential is changed from the unmodified (-10.6 ± 4.2 mV) into the functionalized (30.1 ± 6.3 mV). Meanwhile, arginine-functionalized and europium-doped hydroxyapatite nanoparticles with positive zeta potential can effectively bind negative plasmid DNA, and can be visualized in the cytoplasm and perinuclear of A549 cells by fluorescence microscope and laser scanning confocal microscope.

## Competing interests

The authors declare that they have no competing interests.

## Authors' contributions

ZY and HY conceived and designed the study, carried out the experiments, analyzed the results, and drafted the manuscript. ZJ and LZ assisted in synthesis and characterization of nanoparticles experiments and assisted in cell culture; ZS and ZK supervised the research, contributed in interpretation of data and revision of the manuscript. All the authors have given final approval of the version to be published.
